# Increased demand for paramedic transports to the emergency department in Ontario, Canada: a population-level descriptive study from 2010 to 2019

**DOI:** 10.1007/s43678-022-00363-4

**Published:** 2022-08-19

**Authors:** Ryan P. Strum, Ian R. Drennan, Fabrice I. Mowbray, Shawn Mondoux, Andrew Worster, Glenda Babe, Andrew P. Costa

**Affiliations:** 1 Department of Health Research Methods, Evidence and Impact, McMaster University, Hamilton, ON Canada; 2 Department of Family and Community Medicine, Division of Emergency Medicine, Temerty Faculty of Medicine, University of Toronto, Toronto, ON Canada; 3 Sunnybrook Research Institute, Sunnybrook Hospital, Toronto, ON Canada; 4 Department of Medicine, Division of Emergency Medicine, McMaster University, Hamilton, ON Canada; 5 Institute for Health Policy, Management and Evaluation, University of Toronto, Toronto, ON Canada; 6 Institute for Clinical Evaluative Sciences, McMaster University, Hamilton, ON Canada; 7 Department of Medicine, McMaster University, Hamilton, ON Canada

**Keywords:** Paramedic, Prehospital, Epidemiology, Emergency department, Emergency medical services, Paramédical, Préhospitalier, Epidémiologie, Service des urgences, Services médicaux d'urgence

## Abstract

**Purpose:**

We examined changes in annual paramedic transport incidence over the ten years prior to COVID-19 in comparison to increases in population growth and emergency department (ED) visitation by walk-in.

**Methods:**

We conducted a population-level cohort study using the National Ambulatory Care Reporting System from January 1, 2010 to December 31, 2019 in Ontario, Canada. We included all patients triaged in the ED who arrived by either paramedic transport or walk-in. We clustered geographical regions using the Local Health Integration Network boundaries. Descriptive statistics, rate ratios (RR), and 95% confidence intervals were calculated to explore population-adjusted changes in transport volumes.

**Results:**

Overall incidence of paramedic transports increased by 38.3% (*n* = 264,134), exceeding population growth fourfold (9.4%) and walk-in ED visitation threefold (13.4%). Population-adjusted transport rates increased by 26.2% (rate ratio 1.26, 95% CI 1.26–1.27) compared to 3.4% for ED visit by walk-in (rate ratio 1.03, 95% CI 1.03–1.04). Patient and visit characteristics remained consistent (age, gender, triage acuity, number of comorbidities, ED disposition, 30-day repeat ED visits) across the years of study. The majority of transports in 2019 had non-emergent triage scores (60.0%) and were discharged home directly from the ED (63.7%). The largest users were persons aged 65 or greater (43.7%). The majority of transports occurred in urbanized regions, though rural and northern regions experienced similar paramedic transport growth rates.

**Conclusion:**

There was a substantial increase in the demand for paramedic transportation. Growth in paramedic demand outpaced population growth markedly and may continue to surge alongside population aging. Increases in the rate of paramedic transports per population were not bound to urbanized regions, but were province-wide. Our findings indicate a mounting need to develop innovative solutions to meet the increased demand on paramedic services and to implement long-term strategies across provincial paramedic systems.

## Résumé

**Objectifs:**

Nous avons examiné l’évolution de l’incidence annuelle du transport paramédical au cours des dix années précédant la COVID-19 par rapport à l’augmentation de la croissance de la population et des visites à l’urgence en personne.

**Méthodes:**

Nous avons mené une étude de cohorte au niveau de la population en utilisant le Système national d'information sur les soins ambulatoires du 1er janvier 2010 au 31 décembre 2019 en Ontario, au Canada. Nous avons inclus tous les patients triés aux urgences qui sont arrivés par transport paramédical ou sans rendez-vous. Nous avons regroupé les régions géographiques en utilisant les limites du Réseau local d'intégration des services de santé. Des statistiques descriptives, des rapports de taux (RR) et des intervalles de confiance à 95% ont été calculés pour examiner les variations des volumes de transport ajustées en fonction de la population.

**Résultats:**

L'incidence globale des transports paramédicaux a augmenté de 38.3% (*n* = 264 134), soit quatre fois plus que la croissance démographique (9.4%) et trois fois plus que la fréquentation des urgences sans rendez-vous (13.4 %). Les taux de transport ajustés à la population ont augmenté de 26.2 % (ratio de taux 1.26, IC à 95% 1.26–1.27), contre 3.4 % pour la visite aux urgences sans rendez-vous (ratio de taux 1.03, IC à 95% 1.03–1.04). Les caractéristiques des patients et des visites sont restées constantes (âge, sexe, acuité du triage, nombre de comorbidités, disposition des urgences, visites répétées aux urgences à 30 jours) au cours des années d'étude. La majorité des transports en 2019 avaient des scores de triage non urgents (60.0 %) et ont été renvoyés chez eux directement du service d'urgence (63.7 %). Les plus grands utilisateurs étaient les personnes âgées de 65 ans ou plus (43.7 %). La majorité des transports ont eu lieu dans les régions urbanisées, bien que les régions rurales et du Nord aient connu des taux de croissance du transport paramédical similaires.

**Conclusion:**

Il y a eu une augmentation considérable de la demande de transport paramédical. La croissance de la demande de services paramédicaux a nettement dépassé la croissance de la population et pourrait continuer d’augmenter parallèlement au vieillissement de la population. Les augmentations du taux de transports paramédicaux par population n'étaient pas limitées aux régions urbanisées, mais s'étendaient à l'ensemble de la province. Nos constatations indiquent un besoin croissant d’élaborer des solutions novatrices pour répondre à la demande accrue de services paramédicaux et mettre en œuvre des stratégies à long terme dans l’ensemble des systèmes paramédicaux provinciaux.

## Clinician’s capsule

**Table:** 

***What is known about the topic?***
Little is known if increased demand for Ontario emergency department services extended to paramedic services.
***What did this study ask?***
How do changes in paramedic transports compare to population growth and ED visits by walk-in?
***What did this study find?***
Paramedic transports increased by 38.3%, exceeding population growth fourfold (9.4%) and walk-in emergency department visits threefold (13.4%).
***Why does this study matter to clinicians?***
Paramedic systems should develop innovative care solutions and long-term provincial strategies to manage significant increases in demand for paramedic healthcare.

## Background

Demand for paramedic response and transportation to the emergency department (ED) has increased across Canada [1]. Particular growth in paramedic transports and workload has been reported in Alberta, Nova Scotia, Prince Edward Island and New Brunswick, though little is known about Ontario [2–7]. Ontario’s annual incidence of paramedic transports have yet to be investigated on a population level, despite comprising the largest population (39% of Canada; 14,789,778 persons), second highest provincial growth rate and highest provincial ED visitation rate of all provinces [7, 8]. Conceptually, Ontario’s paramedic transport growth may be similar, or larger, to the sizable increase of ED visits observed between 2008 and 2014, which doubled the populations growth (13.4% versus 6.2%) [9].

Understanding temporal changes in paramedic transports at the population level can improve patient-centered care, and inform future research priorities, health policies, paramedic regulation, and innovative care model development [10]. As highlighted by the additional strain of the COVID-19 pandemic on paramedic services, evaluating the magnitude of paramedic transport growth can help for future system planning, including during unexpected additional stressors, to ensure continued high-quality patient care [11, 12]. Moreover, increasing paramedic transports could be a contributing factor in the growth of ED overcrowding, which impacts various measures of ED performance and quality of care (i.e., time to physician assessment, patient satisfaction, workload) [13–15]. Examination of population-based annual transports could provide a contextual understanding of paramedic demand, allow for inter-provincial comparisons, describe impacts to ED’s and determine whether growth can simply be attributed to provincial population increases. Lastly, studying trends of patient transports with low acuity and non-emergent conditions could support the implementation of new care models for paramedics when patients may not require overstrained ED services.

Our aim was to describe changes in the incidence of paramedic transport to emergency departments in Ontario. Specifically, to examine changes in population growth against changes in ED visitation by paramedic transport and walk-in, as well as to define and describe trends in annual paramedic utilization for transport to the ED.

## Methods

### Study design

We conducted a population-level retrospective cohort study using administrative ED records from the National Ambulatory Care Reporting System (NACRS) database. The reporting of studies conducted using observational routinely collected health data (RECORD) statement was followed for reporting of results [16].

### Population and setting

All patients triaged in an Ontario ED and arrived by either ambulance or walk-in between January 1, 2010 and December 31, 2019 were included. This ten-year timeframe represents the most recently available decade prior to the COVID-19 pandemic, when utilization of paramedic services and ED visitations changed [17]. Examining these data separately avoids contamination of confounding effects and bias related to COVID-19. Only patients arriving at the ED by ground ambulance or walk-in were included to reduce potential selection bias. Paramedic transports by air ambulance or interfacility ground ambulance transfers were excluded as these modes of transport do not signify a patients first healthcare contact occurring in the ED.

### Data source

Data were extracted from the NACRS database, housed in the Institute for Clinical Evaluative Sciences (ICES). NACRS is a hospital and community-based ambulatory care administrative database that collects data of every patient’s ED visit at the time of service in Ontario [18]. Patient visits were identified by isolating ‘location’ in NACRS to the ED and mode of transportation coding to ‘ground ambulance’ and ‘no ambulance arrival (e.g., walk-in)’. All Ontario ED’s provide administrative reports to NACRS quarterly, and no ED was excluded from our study. ICES is a non-profit, independent corporation that supports the study of health service and population-wide outcomes in Ontario using administrative databases. Data of Ontario’s population as a province and for each region were extracted from the Registered Persons Database (RPDB) from ICES. RPDB is a repository of all persons registered in Ontario under the Ontario Health Insurance Plan (OHIP) and eligible for universal healthcare services.

### Variables and outcome measures

Patient age was originally extracted as twenty-two categorical levels due to personal health information privacy restrictions. Age was further collapsed into four categories to parallel provincial figures and prior literature (0–17, 18–39, 40–64, 65–105 years) [1]. Regionalization of Ontario was determined using the location of the ED within fourteen Local Health Integration Network (LHIN) boundaries. Comorbidities were recorded as pre-existing diagnoses at the time of ED visit and included hypertension, diabetes, chronic obstructive pulmonary disease, asthma, rheumatoid arthritis, congestive heart failure, bowel disease, and cancer. Comorbidities included in this study were selected based on data availability. The Canadian Triage and Acuity Scale (CTAS) was used to operationalize triage of patients at entry to ED, assigned by the ED triage nurse, not by a paramedic. CTAS is an ordinal scale that ranges from one to five, with a score of one indicating the highest severity (resuscitation) and five the least urgent (non-urgent) [19]. Non-emergent CTAS sores were considered three (urgent), four (less urgent) and five (non-urgent), indicating no immediate life-sustaining treatment or interventions were required at time of triage [19].

### Statistical analysis

Descriptive statistics were reported using measures of central tendency and frequency. Rate ratios (RR) were computed along with using 95% confidence intervals to adjust for population growth as a predictor of paramedic transport growth [20]. Data were managed and analyzed in R software (v. 3.6) [21]. Missing data were reported directly.

### Ethics approval

ICES’s collection and use of NACRS secondary ambulatory data is authorized under Section 45 of Ontario’s Personal Health Information Protection Act (PHIPA) as a prescribed entity, which is exempt from review by a Research Ethics Board [22, 23]. The use of the data in this study is authorized under Section 45 and approved by ICES’s Privacy and Legal Office.

## Results

**Fig. 1:**
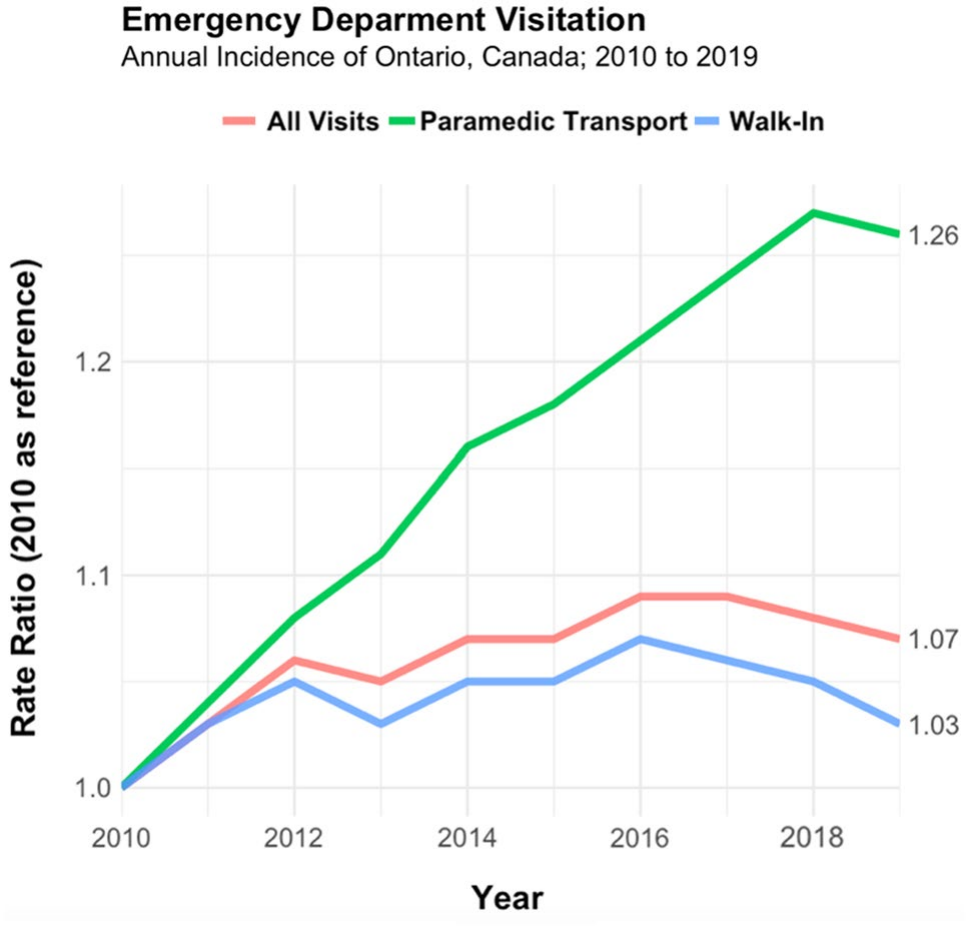
Emergency department visitation growth rate by mode of transportation from January 1, 2010 to December 31, 2019 in Ontario, Canada

Ontario’s paramedic transports increased by 38.3% (264,134) during the ten-year study period, the provincial population increased by 9.4% (1,240,057), and ED visits by walk-in increased by 13.4% (582,854). Table 1 shows the population-adjusted rate ratios of ED visits by mode of arrival per 100,000 Ontarians. Paramedic transported patients increased by 1,384 transports per 100,000 Ontarians (5,246 in 2010 to 6,630 in 2019), constituting a rate ratio of 1.26 (95% CI 1.26–1.27). Paramedic transports increased considerably compared to walk-in (RR 1.03, 95% CI 1.03–1.04) or the overall ED visitation (RR 1.07, 95% CI 1.07–1.08). Growth of Ontario’s ED visitation rate ratios by mode of transport are shown for 2010–2019 in Fig. 1. Strong linearity was observed in paramedic transport growth across all years until 2018. Total ED visits and ED visits by walk-in had variable growth until 2016, with slight decreases each year until 2019.

**Table 1:** Rate ratios per 100,000 Ontarians of ED visitation by paramedic utilization and ED walk-in from 2010 to 2019 in Ontario, Canada

	Annual incidence, *n*			Incidence rate per 100,000 Ontarian’s		
	2010	2019	Increase (%)	2010	2019	Rate ratio (95% CI)
Population	13,142,241	14,382,298	1,240,057 (9.4)	–	–	–
Total ED Visitation	5,047,149	5,984,137	946,988 (18.6)	38,404	41,608	1.07 (1.07–1.08)
Paramedic transport	689,479	953,613	264,134 (38.3)	5,246	6,630	1.26 (1.26–1.27)
Walk-in	4,357,670	4,940,524	582,854 (13.4)	33,158	34,351	1.03 (1.03–1.04)

Patient and visit characteristics were consistent between 2010 and 2019. The older age cohort (65–105 years) represented the largest group of paramedic transports and remained consistent from 2010 to 2019 (42.4 and 43.7%). The young adult population (18–39 years) had the largest population-adjusted rate ratio increase per 10,000 Ontarians (RR 1.35, 95% CI 1.34–1.36), indicating the largest growth of paramedic transports by this age cohort.

Table 2 displays a detailed list of patient demographic and visit characteristics, alongside their rate ratios after population adjustment. The majority of patients transported by paramedics had non-emergent CTAS acuity scores (60.0% in 2019). Over half of all paramedic transports were in the urgent triage acuity CTAS score (CTAS 3) in the study years. The least severe triage acuities comprised the largest rate ratio (RR 2.2, 2.2–2.3), though only constituted approximately 1% of transports. Nearly two-thirds of patients transported were discharged from the ED (63.7%), a result consistent with 2010 (63.5%). The majority of patients transported did not return to the ED within 30 days (73.3%). The number of comorbidities greatly influenced the growth in need for paramedic transport, with persons having six or more conditions having a two-fold increase in paramedic service use growth, when compared to those with zero–two conditions (RR 1.5 versus 1.2, respectively). No missingness was reported for any variable.

**Table 2:** Characteristics of paramedic transported emergency department visits between 2010 and 2019 in Ontario, Canada

Characteristics	2010, *n* (%)	2019, *n* (%)	Rate ratio increase^c^ (95% CI)
Age, years			
0–17	82,830 (12.0)	97,837 (10.3)	1.1 (1.1–1.1)
18–39	109,430 (15.9)	161,315 (16.9)	1.4 (1.3–1.4)
40–64	204,051 (29.6)	277,453 (29.1)	1.2 (1.2–1.3)
65–105	293,168 (42.4)	417,008 (43.7)	1.3 (1.3–1.3)
Gender			
Male	316,401 (45.9)	450,101 (47.2)	1.3 (1.3–1.3)
Female	373,078 (54.1)	503,512 (52.8)	1.2 (1.2–1.2)
Triage Acuity, CTAS			
Resuscitation (1)	19,292 (2.8)	36,899 (3.9)	1.8 (1.7–1.8)
Emergent (2)	234,129 (34.0)	343,591 (36.0)	1.3 (1.3–1.4)
Urgent (3)	353,365 (51.3)	484,660 (50.8)	1.3 (1.3–1.3)
Less Urgent (4)	77,039 (11.2)	76,115 (8.0)	0.9 (0.9–0.9)
Non-Urgent (5)	4,601 (0.7)	11,237 (1.2)	2.2 (2.3–2.3)
Triaged but not Reported	1,053 (0.0)	1,111 (0.0)	–
Comorbidities^a^			
0–2	514,890 (74.7)	693,612 (72.7)	1.2 (1.2–1.2)
3–5	171,061 (24.8)	254,051 (26.6)	1.4 (1.4–1.4)
6–8	3,528 (0.5)	5,950 (0.6)	1.5 (1.5–1.6)
ED Visit Disposition			
Discharged from ED	437,896 (63.5)	607,768 (63.7)	1.3 (1.3–1.3)
Admitted to Hospital	223,186 (32.3)	298,155 (31.3)	1.2 (1.2–1.2)
Other	28,397 (4.2)	47,690 (5.0)	1.5 (1.5–1.6)
Returned to ED within 30 days			
Yes	156,132 (22.6)	253,760 (26.6)	1.5 (1.5–1.5)
No	533,347 (77.4)	699,853 (73.3)	1.2 (1.2–1.2)

*CTAS * Canadian Acuity and Triage Scale, *ED* emergency department

^a^Total of comorbidities present on ED arrival, included: hypertension, diabetes, chronic obstructive pulmonary disease, asthma, rheumatoid arthritis, congestive heart failure, bowel disease, cancer

^b^Determined using forward sortation area (FSA) postal codes in Ontario

^c^Per 100,000 Ontarians

**Fig. 2:**
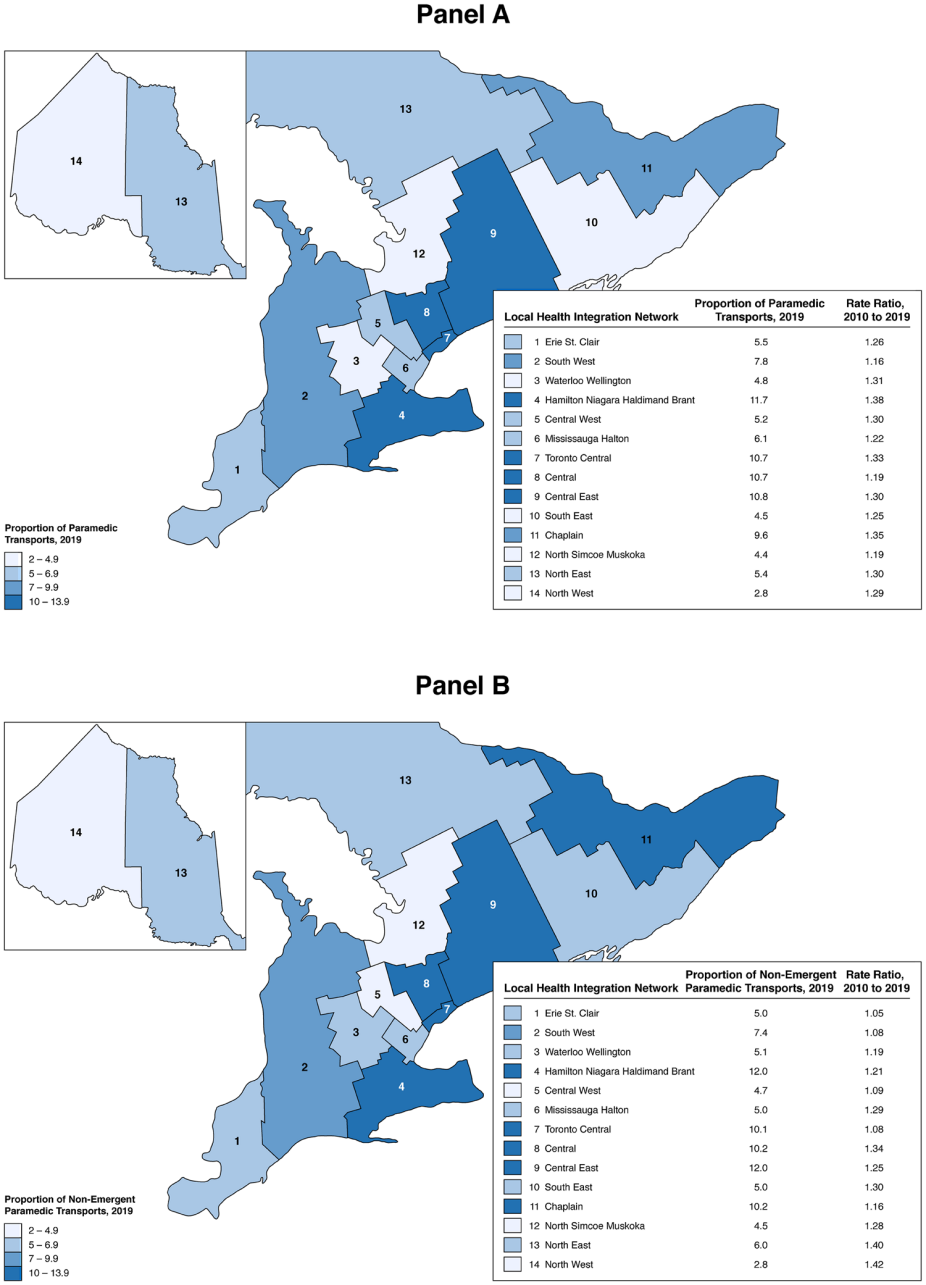
Map of paramedic transported patients using Local Health Integration Network boundaries in Ontario, Canada for rate ratios of population-adjusted increases in transports per 100,000 Ontarians between 2010 and 2019. Panel **A** Proportion of paramedic transports in 2019. Panel **B** Proportion of non-emergent triaged paramedic transports in 2019

Incidence and rate ratios of all paramedic transported patients in Ontario by LHIN region are shown in Fig. 2. In the most current year of study (2019), the incidence of paramedic transported patients differed across geographical LHIN regions of Ontario. The largest proportion of paramedic transports occurred in LHIN regions south and central in Ontario, encapsulating the majority of urbanized regions (53.4%). Hamilton Niagara Haldimand Brant (11.7%), Central East (10.8), Toronto Central (10.7%) and Central (10.6%) LHIN’s composed the highest proportion of Ontario’s transports, while North West (2.4%) and North Simcoe Muskoka (4.4) denoted the least. Population-adjusted rate ratios per 100,000 Ontarians per LHIN were computed to examine changes in paramedic transports relative to each LHIN region. We identified an increase in paramedic transports across all Ontario LHIN regions (RR range 1.16–1.38). The largest rate ratios were not specified to regions of higher numbers of paramedic transports, but dispensed throughout the province. In an exploration of non-emergent transports, similar proportion of transports was observed across LHIN regions; however, larger rate ratios were observed in the rural LHIN regions.

## Discussion

### Interpretation of findings

Population-level incidence rates of paramedic transports increased during the ten-year timeframe of our study. Paramedic transports far outpaced population growth and ED visitation by walk-in, with most patients being 65 years or older, assigned an urgent triage acuity, and discharged directly from the ED. Overall, rates of paramedic transports increased across all variables measured in this study except for transport with triaged as CTAS 4. Ontario’s largest proportion of paramedic transports occurred in urbanized regions, though equally large population-adjusted increases were observed in rural and northern regions.

### Previous studies and reports

Ontario’s growth in paramedic transport is equal to or greater than the growth observed in other provinces, but at a significantly higher crude annual volume [2–5]. The reason for increased rates of paramedic transports is complex and likely multifactorial. We speculate some of the contributing factors include: difficulty in accessing primary healthcare, a lack of timely access to care, perceived situation by the patient, a sense of superior in-hospital care, and a lack of awareness of other healthcare services [24–26]. Consistent with previous literature, we found that older age groups and increasing comorbidities may be contributing factors to increased paramedic utilization [6, 27, 28].

Annual paramedic transports increased at a consistent rate, year to year, from 2010 to 2018, with a minimal decrease in 2019 likely due to the COVID-19 pandemic effects on the later months [17, 29]. The lack of variance in annual transport incidence as well as the expanding number of older adults, whom are the largest users of paramedic and health services, suggests transport volumes will continue to grow [30]. The lack of published population-adjusted analyses on paramedic transport rates in Canada limits direct comparisons to our study, and constitutes a gap in the literature.

### Strengths and limitations

To our knowledge, this is the first study to examine changes in the cumulative incidence of paramedic transports to the ED at a population-level in Ontario, Canada. We used population-level data from Ontario, representing approximately 40% of all Canadians. Our population-level adjusted analyses are likely generalizable to other provinces in Canada, where crude increases in paramedic utilization have also been reported.

Overall paramedic utilization is not represented in this study given only patients transported to an ED could be included from ED data sources. Paramedic transport data are not readily available for population-level analyses. Additionally, there are fundamental limitations associated with administrative databases, as we were only able to analyze a limited set of variables, which did not support the inclusion of patient participation in our analyses.

### Health policy and regulation implications

Contrary to traditional conceptualizations of paramedicine as an emergency service, the majority of paramedic transported patients have non-emergent medical complaints and conditions [1, 6]. Expanding and aligning paramedic scope of practice, clinical decision-making, and models of care with primary care skills and service accessibility may support efforts to improve patient-centered care and lessen the burden of ED workload [1, 31].

Our findings are important in supporting long-term strategies for paramedic services, hospitals, and regulatory institutions to address the growing demand for paramedics. Actionable solutions and implementation of new models of care by paramedic services are required to address increased paramedic workloads, especially when increases in paramedic staffing, organizational structures and ambulances do not address the fundamental burden of increased demand and are slow. These results showcase the opportunity paramedic services and stakeholders have to step into new roles within healthcare for paramedics. Redirection of specific patients to subacute and community-based providers could be a valuable strategy to decrease ED workloads when these may offer similar or equivalent care to the ED when patient conditions are non-emergent and largely require primary care or greater assessment time [32–34]. Conceptually, paramedic transport to subacute care centers is a practical solution to offset ED visits when as they may provide applicable care alternatives while providing higher cost-effectiveness for less urgent complaints, and could provide care with reduced waiting times [32–34].

Expanding and refining paramedic strategies used nationally and internationally such as secondary triage, virtual care, nurse practitioner outreach, community paramedicine and paramedic treat-and-release programs may also address ongoing paramedic demand surges, though further investigation is required to understand the effectiveness of these strategies [35–38]. Additionally, programs that aim to reduce paramedic usage for repeated events could be practical and advantageous to reduce 911 calls, such as opioid overdose-related transports [39]. Programs aimed at increasing optimization of ambulance availability may also be constructive, such as decreasing offload delay at hospital transfer of care [40].

### Research implications

The findings of this study highlight the need for future research on non-traditional paramedic scopes of practice. Research is required to better understand the needs of patients who call 911 to align future clinical scope of practice and development of alternative solutions to patient transport to the ED. Understanding patient rationales for calling paramedics could support the development of decision-support tools to advise when to call for paramedics. Development and implementation of standardized provincial paramedic reporting systems would allow for inter-provincial collaboration and comparison of paramedic system utilization, and aid researchers to better analyze population-level factors that influence paramedic utilization on an ongoing basis. Other Canadian provinces could benefit from similar population-adjusted research to better understand utilization trends of their paramedic systems.

## Conclusion

Paramedic patient transport to the ED in Ontario, Canada increased significantly and steadily in the ten years prior to the COVID-19 pandemic. The transport rate increased fourfold compared to population growth, and threefold compared to walk-in ED visit rates. Paramedic services may be unable to maintain timely and high-quality service without a concomitant capacity increase if the trends in demand continue. Our findings support discussions on the need to consider secondary triage and alternate transport models to cope with increasing service demands.

## Data Availability

All aggregate data herein are accessible to other interested parties by application to the corresponding author.
